# Assessment of childhood pneumonia management and outcomes at primary healthcare centres in Islamabad and Rawalpindi: an observational cohort study from Pakistan

**DOI:** 10.7189/jogh.16.04137

**Published:** 2026-05-22

**Authors:** Hana Mahmood, Azka Hafeez, Aaliyan Kashif, Syed Yahya Sheraz, Memoona Shehzadi, Shamim Ahmad Qazi, Yasir Bin Nisar

**Affiliations:** 1 Research Department, International Research Force, Islamabad, Pakistan; 2 Operations Department, International Research Force, Islamabad, Pakistan; 3 Data Department, International Research Force, Islamabad, Pakistan; 4 Independent consultant, Geneva, Switzerland; 5 Department of Sexual, Reproductive, Maternal, Child, Adolescent and Ageing Health (LHR), World Health Organization, Geneva, Switzerland

## Abstract

**Background:**

Pneumonia remains a leading cause of under-five mortality in Pakistan. The Government of Pakistan adopted the revised World Health Organization guidelines, which recommend outpatient management of chest indrawing pneumonia using oral amoxicillin in children aged 2–59 months. We evaluated the management and outcomes of the guidelines at the primary healthcare (PHC) settings in Islamabad and Rawalpindi, Pakistan.

**Methods:**

We conducted a prospective observational cohort study (January 2024–February 2025) across seven PHCs. Children aged 2–59 months with cough and/or difficult breathing and chest indrawing without general danger signs, who lived in the catchment area, were enrolled following caregiver consent and followed up on day 15. The primary outcome was the case fatality rate (CFR), with secondary outcomes being caregiver-reported cure, hospitalisation, and antibiotic adherence in follow-up children.

**Results:**

Of 3358 children screened, 373 (11.1%) were diagnosed with chest indrawing pneumonia. Median age was 11 months, and 57.1% (n = 213) were male. By day 15, 366 children were successfully followed up. The CFR was 0.6% (n = 2; 95% confidence interval (CI) = 0.1–2.1), with both deaths in infants (aged 2–11 months). Caregivers reported that 89.3% (n = 327) were cured, 9.0% (n = 33) remained unchanged, and 1.1% (n = 4) worsened. Further, 12 children (3.3%) were hospitalised. At enrolment, 93.8% (n/N = 350/373) were prescribed oral antibiotics, predominantly amoxicillin (n = 322, 92.0%). Moreover, of these 350 children, 307 (87.7%) initiated treatment at home. Within this subgroup, 90.2% (n = 277) completed the prescribed course, and 89.9% (n = 249) of those completing treatment were cured. Among those who did not complete treatment, one child worsened, and another died.

**Conclusions:**

Outpatient treatment of chest indrawing pneumonia with oral amoxicillin at the PHC level is safe and effective, with low mortality and high cure rates. Strengthening Integrated Management of Childhood Illnesses protocols and supervision can reduce hospital referrals, while timely care-seeking and caregiver adherence can further improve outcomes in Pakistan and similar low-resource settings.

Pneumonia is an acute infection of the lungs and continues to be one of the leading causes of under-five mortality globally [1]. In 2023, 4.8 million children died before reaching the age of five [2]. The median rate of clinical pneumonia in children aged <5 years in low- and middle-income countries (LMICs) is estimated at 0.29 episodes per child year [3]. Although between 2000 and 2015, there was a 51% reduction in pneumonia-related deaths globally, it is still one of the leading causes of death in this age group [4]. It is essential to note that this burden is disproportionately borne by children residing in Sub-Saharan Africa and South Asia, where access to necessary health services remains limited. Nearly a third of all under-five deaths due to pneumonia have occurred in just five countries: Nigeria (n = 162 000), India (n = 127 000), Pakistan (n = 58 000), the Democratic Republic of the Congo (n = 40 000), and Ethiopia (n = 32 000) [5,6]. Pakistan, the world’s sixth most populous nation, has an estimated population of 35.3 million children aged <5 years, and an estimated seven deaths per 1000 live births are attributed to pneumonia [7,8].

In 2012, the World Health Organization (WHO) updated pneumonia management guidelines and introduced outpatient treatment with oral amoxicillin for children aged 2–59 with chest indrawing pneumonia, but without general danger signs [9,10]. For outpatient therapy in primary healthcare facilities, the WHO and the United Nations International Children’s Emergency Fund (UNICEF) developed the Integrated Management of Childhood Illness (IMCI) strategy, which includes assessment, classification, and treatment guidance for common childhood illnesses, such as pneumonia [11]. For inpatient treatment in small or district hospitals, the WHO Pocket Book of Hospital Care for Children is the main reference book on the management of childhood pneumonia and other serious diseases [10]. Further, 45 countries have adopted the outpatient management of pneumonia; however, real-world evidence of its effectiveness remains limited [12].

In 2018, a retrospective analysis of hospitalised children with pneumonia in Kenya reported significantly higher mortality among those presenting with chest indrawing, particularly when accompanied by mild to moderate palmar pallor and a weight-for-age Z score (WAZ) below –3 standard deviations [13]. These findings raised concerns among experts [14,15]. The WHO held a meeting in 2018 to review published and unpublished evidence on mortality risks in children with chest indrawing pneumonia [16]. It was recommended to collect real-world evidence on the outcomes of outpatient treatment for children with chest indrawing pneumonia, in line with the revised WHO guidelines.

In this study, we collected outcome data for children aged 2–59 months with chest indrawing pneumonia who presented at selected primary healthcare (PHC) facilities in the Islamabad and Rawalpindi districts of Pakistan. The selected sites offer a strong and informative context for evaluating outpatient pneumonia management, as they encompass a mix of urban, peri-urban, and rural communities, are supported by a well-established PHC network, and report some of the highest caseloads of acute respiratory infections. The primary outcome was the case fatality rate (CFR) at day 15 post-enrolment, and secondary outcomes included caregiver-reported cure rates, hospitalisation, and antibiotic adherence.

## METHODS

### Study design and setting

This prospective observational cohort study was part of a multicentre, multi-country initiative conducted under the Chest Indrawing Pneumonia Management (CIPAM) Study Group, which aimed to assess the management and outcomes of children aged 2–59 months with chest indrawing pneumonia presenting at PHC facilities across six countries: Ethiopia, India, Nigeria, Pakistan, Uganda, and Zambia [17]. In Pakistan, this study was conducted at three sites – Thatta, Lahore, and Islamabad/Rawalpindi. In this manuscript, we present findings from the Islamabad/Rawalpindi site, where we selected seven PHCs based on the highest caseloads of acute respiratory infections (ARI) identified in the district health information systems data for the Islamabad and Rawalpindi, serving a combined catchment population of approximately 384 000 people. The health facilities were the Rural Health Centre (RHC) Bhara Kahu, RHC Sihala, RHC Tarlai, Community Health Centre Rawat, and Basic Health Unit (BHU) Sohan in Islamabad, as well as BHU Pind Nosheri and BHU Brehma in Rawalpindi district. They were selected based on consistently high ARI caseloads reported in the district health information system. This selection approach was aligned with the multicountry CIPAM study protocol, which required including sites with sufficient patient flow to ensure feasible recruitment and timely attainment of the target sample size [17]. High-caseload primary healthcare facilities were therefore considered the most appropriate settings for evaluating the real-world implementation of outpatient management of chest indrawing pneumonia within routine service delivery (Figure S1 in the **Online Supplementary Document**)

### Participants

We screened children aged 2–59 months who presented at a selected PHC facility with cough and/or difficulty breathing. Further, we included in the study children aged 2–59 months who were residing in an area where day 15 follow-up assessments could feasibly be conducted, were seeking care at the selected PHC facility for cough and/or difficulty breathing and chest indrawing, and whose caregivers consented to participate. We excluded children if they were aged <2 months or >5 years, had an oxygen saturation below 90%, had stridor when calm, or had any general danger signs (convulsions, inability to drink or feed, vomiting everything, lethargy or unconsciousness) [11]. Furthermore, we excluded children who were temporarily visiting the locality or were currently enrolled in another study.

### Study procedures

#### Preparatory phase

Before initiating enrolment, we obtained the necessary administrative permissions from the Islamabad and Rawalpindi district health offices and other relevant authorities. Furthermore, we obtained ethics clearance. Data collection instruments, including an initial assessment form, a follow-up form, and a verbal autopsy form (in the event of death), were migrated to a tablet-based platform.

#### Training

We conducted a two-day training session for the project staff, covering study objectives, tablet use for data collection, consent procedures, data collection protocols, and simulated interviews. Additionally, the healthcare providers (HCPs) at the study sites received an initial one-day training focused on pneumonia case diagnosis and IMCI management. Later, we organised a four-day reinforcement training for the HCPs to refresh key study concepts. Project staff received ongoing refresher training and on-site guidance during supervisory visits, where discussions with the HCP and project staff were routinely held to ensure adherence to protocol. To ensure consistency across data collectors, standardised training was provided, followed by harmonisation sessions. Supervisors conducted periodic on-site observations and cross-checked randomly selected records from each PHC. Although formal inter-rater reliability statistics were not computed, these procedures ensured standardised assessment and data entry across facilities.

#### Pilot study

Following the training, we conducted a pilot study from 1 December 2023 to 20 January 2024 to assess caseloads, the usability of the collection tools, and the monitoring of healthcare practices. Following the results of the pilot study, we made necessary adjustments to the tools and procedures and provided further refresher training to research and healthcare staff to ensure consistency before the main study commenced on 22 January 2024.

#### Screening and enrolment procedures

**Figure 1:**
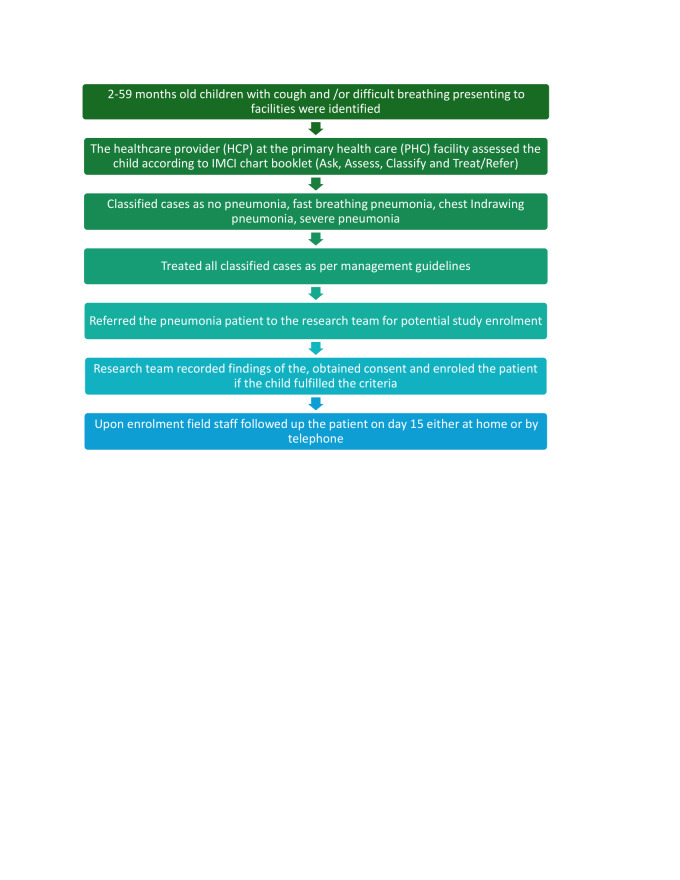
Flowchart representing the screening, selection, and data collection process. HCP – healthcare provider, IMCI – integrated management of childhood illness, PHC – primary healthcare.

The HCPs at the PHCs screened all children aged 2–59 months presenting with ARI symptoms. They obtained their histories, examined them, classified them, and treated them (**Figure 1**). HCPs referred those diagnosed with chest indrawing pneumonia to the research staff at the same facility for potential enrolment in the study. The study research staff assessed the referred children against the study criteria, obtained consent from the caregivers of eligible children, enrolled them, and assigned each enrolled child a unique study identifier. We gave a card to the caregiver to facilitate a follow-up on day 15. It included the unique identifier, the patient’s name, the father’s name, the date of follow-up, and the research staff’s contact details. All data in the assessment form was uploaded to the server and accessed daily by the data officer to ensure completeness and quality and to develop a roster for field team follow-ups.

#### Follow-up and outcome assessment

Upon receiving the follow-up roster, the supervisor developed a comprehensive field plan and assigned specific locations to the designated field staff. The designated field staff called the caregiver to arrange a follow-up appointment on day 15. At the caregiver’s convenience, the follow-up interview was conducted by telephone or face-to-face during a home visit. Information gathered in the interviews was entered into tablets and later uploaded to the central server. The data officer periodically reviewed the data entries to ensure completeness and adherence to quality standards. As this was an observational study embedded in routine PHC service delivery, children were not re-examined by clinicians at follow-up. Caregiver-reported cure was therefore accepted as the primary indicator of recovery. However, interviewers used structured probing questions on persistent symptoms (fever, difficulty breathing, feeding) to minimise misclassification and improve the reliability of caregiver reports.

We defined loss to follow-up under the following circumstances: if the caregiver was unreachable for three consecutive days despite repeated calls, if the phone remained switched off for three consecutive days, if upon contact it was found that the family had shifted away from the city and did not wish to give an interview over the phone, or if the caregiver refused to have the follow-up done. Additionally, if the follow-up was extended beyond day 18 for any reason, it was also considered a loss to follow-up.

At follow-up, if any death was identified, a structured verbal autopsy interview was conducted with caregivers using a standardised tool within six weeks of death to explore the possible causes and contributing factors [18].

#### Sample size

Utilising the standard formula for a single proportion, and based on a 5% case fatality ratio for chest indrawing (derived from primary care data obtained from Malawi; personal communication with Humphrey Nsona on 21 September 2021), a 3% margin of error, and a 95% confidence level, the sample size required for descriptive analysis was computed to be 292 for each site. To account for an anticipated 5% loss to follow-up, a total of 310 children, aged 2–59 months and diagnosed with chest indrawing pneumonia, were enrolled and monitored across all sites [17].

#### Data analysis

We entered and cleared the data in Microsoft Excel (Microsoft Corporation, Redmond, Washington, USA), and all statistical analyses were conducted in Stata, version 17 (StataCorp LLC, College Station, Texas, USA). We generated descriptive statistics to summarise baseline demographic and clinical characteristics of enrolled children. We reported categorical variables such as sex, health facility attended, type of antibiotic prescribed, and treatment outcomes as frequencies and percentages. We summarised continuous variables such as age using means and standard deviations (SDs) or medians and interquartile ranges, depending on the distribution.

Treatment outcomes were classified as ‘cured,’ ‘same,’ ‘worsened,’ or ‘died,’ and the proportions were calculated with corresponding 95% confidence intervals (CI). Anthropometric markers, including weight-for-age (WAZ), weight-for-length (WLZ), and height-for-age (HAZ) Z-scores, were derived from the WHO growth standards [19]. Antibiotic adherence was described as an outcome and compared using cross-tabulations. Additional treatment interventions, such as referrals, admissions, or injections, were also tabulated.

We summarised descriptively findings from the verbal autopsy interviews, focusing on the sequence of events, reported symptoms before death, care-seeking behaviour, and any delays in treatment. The causes of death were inferred based on symptom patterns and contextual information.

## RESULTS

**Figure 2:**
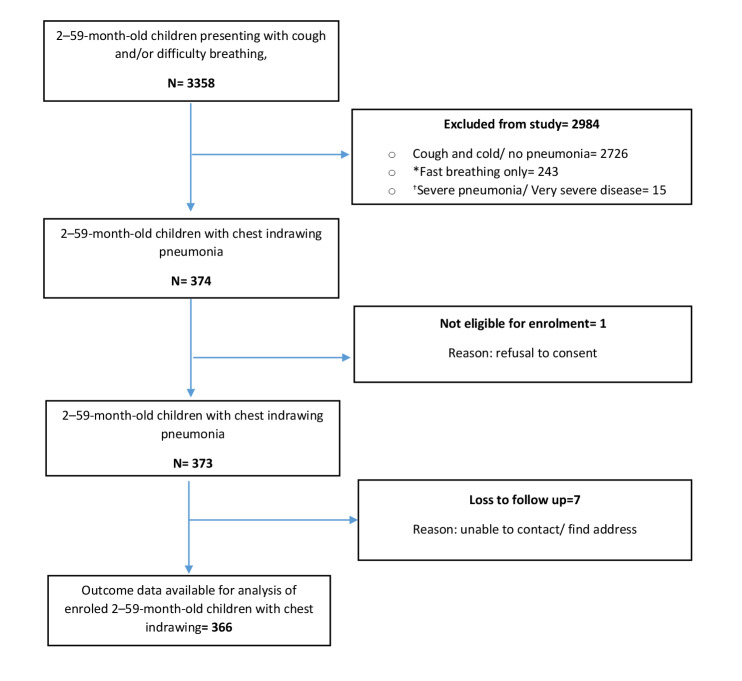
Flowchart of ARI case screening, exclusions, and final enrolment. *Fast breathing – if respiratory rate is 50 breaths per minute or more in 2–11 months and 40 breaths per minute or more in 12–59-month old children. †Severe pneumonia is defined as the presence of any danger signs with pneumonia.

From 22 January 2024 to 15 February 2025, we screened 3358 children aged 2–59 months presenting with cough and/or difficulty breathing. Of that, 2726 had only cough and cold (no pneumonia), 243 had fast-breathing pneumonia, 15 had severe pneumonia, and 374 children had chest indrawing pneumonia. Of these 374 children, one caregiver refused to give consent; thus, 373 children were enrolled. After excluding seven losses to follow-up, the day 15 outcome data were available for 366 children with chest indrawing pneumonia (**Figure 2**).

### Baseline clinical characteristics

Among the 373 enrolled children, 213 (57.1%) were male, 188 (50.4%) were aged 2–11 months, and the mean age was 15.5 months (SD = 13). Further, 35 (9.4%) children had temperatures ≥38°C and 288 (77.2%) had oxygen saturation between 94–100%. Further, 112 (30.0%) had only chest indrawing, and 261 (70.0%) also exhibited fast breathing, accompanied by chest indrawing (**Table 1**). Of the total participants, the majority, 132 (35.3%), were enrolled from RHC Tarlai, followed by 72 (19.3%) from BHU Sohan. At the time of enrolment, the majority (n = 350; 93.8%) were prescribed oral antibiotics by the HCP, 11 (2.9%) were advised home care, and 12 (3.2%) were referred to tertiary care hospitals for further management. The anthropometric measurements showed that 17 (4.6%) children had WAZ<–3, 37 (9.9%) had WLZ<–3, and 52 (13.9%) had HAZ<–3.

**Table 1:** Baseline characteristics of children aged 2–59 months with chest indrawing pneumonia (n = 373)

Characteristics	n (%)
Health facility	
*BHU Brehma*	32 (8.7)
*BHU Pind Nosheri*	13 (3.5)
*BHU Sohan*	72 (19.3)
*CHC Rawat*	46 (12.3)
*RHC Bhara Kahu*	45 (12.0)
*RHC Sihala*	33 (8.9)
*RHC Tarlai*	132 (35.3)
Age in months	
*x̄ (SD)*	15.5 (13.0)
*MD (IQR)*	11.0 (6.0–23.0)
Age categories in months	
*2–11*	188 (50.4)
*12–59*	185 (49.6)
Sex	
*Male*	213 (57.1)
*Female*	160 (42.9)
Baseline distribution of chest indrawing cases	
*Chest indrawing only*	112 (30.0)
*Chest indrawing with fast breathing*	261 (70.0)
Chest indrawing (n = 112)*	
*2–11 months*	55 (49.1)
*12–59 months*	57 (50.9)
Chest indrawing with fast breathing (n = 261)†	
*2–11 months*	133 (51.0)
*12–59 months*	128 (49.0)
Axillary temperature in °C	
*x̄ (SD)*	37.0 (0.6)
*MD (IQR)*	36.6 (36.6–37.2)
Temperature category in °C	
*<38*	338 (90.6)
*≥38*	35 (9.4)
Respiratory rate breaths per minute (age 2–11 months)	
*x̄ (SD)*	51.6 (7.5)
*MD (IQR)*	52.5 (46.3–56.5)
Respiratory rate breaths per minute (age 12–59 months)	
*x̄ (SD)*	43.8 (9.1)
*MD (IQR)*	43.5 (38.5–48.5)
Oxygen saturation in %	
*x̄ (SD)*	96.5 (3.4)
*MD (IQR)*	98.00 (95.0–99.0)
Oxygen saturation category	
*90–93*	85 (22.8)
*94–100*	288 (77.2)
Management prescribed at enrolment	
*Home care, no antibiotics*	11 (2.9)
*Oral antibiotics on an outpatient basis*	350 (93.8)
*Hospital referral with a pre-referral dose*	10 (2.7)
*Hospital referral without a pre-referral dose*	2 (0.5)
Adult primary respondent (n = 366)‡	
*Mother*	165 (44.2)
*Father*	173 (46.4)
*Grand mother*	5 (1.3)
*Other§*	23 (6.2)
*Missing¶*	7 (1.9)
WAZ score	
*<–3*	17 (4.6)
*–3≤WAZ≤–2*	44 (11.8)
*>–2*	312 (83.6)
WLZ score	
*<–3*	37 (9.9)
*–3≤WLZ≤–2*	64 (17.2)
*>–2*	272 (72.9)
HAZ score	
*<–3*	52 (13.9)
*–3≤WAZ≤–2*	43 (11.5)
*>–2*	278 (74.3)
MUAC in cm	
*≥12.5*	364 (97.6)
*11.6–12.5*	7 (1.9)
*≤11.5*	2 (0.5)

BHU – basic health unit, CHC – community health centre, HAZ – height-for-age-z-score, IQR – inter-quartile range, MD – median, MUAC – mid-upper arm circumference, RHC — rural health centre, SD – standard deviation, WAZ – weight-for-age-Z-score, WLZ – weight-for-length-Z-score, x̄ – mean

*Chest indrawing pneumonia cases only.

†Chest indrawing with fast breathing pneumonia.

‡Number of successfully followed-up cases of chest indrawing.

§Aunt, grandfather, and uncle of the enrolled patient.

¶Children lost to follow-up.

### Primary outcome

Two deaths were reported among the 366 followed-up children, yielding a CFR of 0.6% (95% CI = 0.1–2.1). The overall mortality incidence was 0.4 deaths per 1000 child-days. Both deaths occurred among infants aged 2–11 months. Of the 366 children who were followed up, based on caregivers’ reports, 327 (89.3%) were cured, 33 (9.0%) remained the same, and 4 (1.1%) were worsened (**Box 1**, **Table 2**; Table S1 in the **Online Supplementary Document**).

Box 1 **Verbal autopsy summaries of two deaths**

**Verbal autopsy death one: **on 31 December 2024, a six-month-old female child was brought to the Community Health Centre Rawat with fever, difficulty breathing and cough. The mother described noisy sounds coming from the child's chest. The child's initial symptoms, fever and cough, began on 29 December 2024. The child was fully immunised for her age. Her weight was eight kg, length was 67 cm, temperature was 37.2°C, respiratory rate was 64/min, pulse oximetry was 98%, and C-reactive protein level was 40–80 mg/L. The child was diagnosed with chest indrawing pneumonia by the healthcare provider, who prescribed oral amoxicillin for five days at home. However, the mother was concerned and requested a referral, which was subsequently made to a public-sector tertiary hospital in Rawalpindi. She was admitted on the same day. Before going to the hospital, the mother bought oral clarithromycin from a local pharmacy on her own. In the hospital, the child was given intravenous medicine (unknown) for three days while the mother continued oral clarithromycin simultaneously. A chest radiograph was performed. The mother reported that the doctor had told her the chest radiograph was normal, but her blood tests showed an elevated white blood cell count and a red blood cell count below the normal range. The child was discharged after three days because her fever had settled. The mother did not want the discharge because her child had not recovered fully, but the father wanted them to go home. The next morning, the child did not wake up at her usual feeding time at six am. She was woken up at nine am, but she had not drunk formula milk and was very lethargic. The mother placed the child in sunlight on her bed, and the child became a bit more active. She also drank a little milk. Her grandmother took her to get ‘dum’ (traditional spiritual healing) from a known spiritual healer. That afternoon, the child started vomiting (blackish-coloured liquid) and became unresponsive. The child was taken to a private doctor at five pm. On the way, the child was unresponsive, very pale, and water was coming out of her mouth. The child had 39.4°C temperature, and the doctor said the child’s brain was affected and advised caregivers to take the child to a tertiary care hospital immediately. The child passed away at 9 pm on the way to the hospital and was declared dead upon reaching the hospital. **Verbal autopsy death two:** A three-month-old child had a history of fever and cough and was brought to Rural Health Centre Tarlai on 16 January 2025, the third day of illness. The child weighed 4.5 kg, measured 59 cm, had a body temperature of 36.6°C, a respiratory rate of 70 breaths per minute, pulse oximetry was 93%, and a C-reactive protein <10 mg/l. The child had only received one dose of vaccination at birth. The child was diagnosed with chest indrawing pneumonia and prescribed oral amoxicillin by the healthcare provider and sent home. However, the treatment was stopped on day two of therapy because the child did not improve. On the third day after the visit to the study PHC facility, the child was taken to a tertiary care hospital where he was admitted, and intravenous medication (unknown) was started. According to the respondent, the nurses instructed the mother not to breastfeed the child, but she continued breastfeeding secretly when she visited the child. The doctors suspected a heart problem and performed an echocardiogram. The child had difficulty breathing and was given oxygen. On the fourth day of admission, his condition deteriorated, and he was placed on life support. He became unconscious, developed limb swelling, and passed away on 23 January 2025 while on the ventilator. No hospital records were found with the family. The child was operated on for a congenital hernia in the first month of life.

**Table 2:** Outcomes of children with chest indrawing pneumonia who were followed up by age categories (n = 366)

Outcome	Total (n = 366)	2–11 months (n = 186)		12–59 months (n = 180)	
	**n (%)**	**n (%)**	**95% CI**	**n (%)**	**95% CI**
Cured	327 (89.3)	163 (87.6)	82.0–91.6	164 (91.1)	85.0–94.0
Same	33 (9.0)	19 (10.2)	6.5–15.5	14 (7.8)	4.6–12.0
Worse	4 (1.1)	2 (1.1)	0.2–4.2	2 (1.1)	0.2–4.3
Died	2 (0.6)	2 (1.1)	0.2–4.2	NA	NA

CI – confidence interval, NA – not available

### Secondary outcomes

Of 366 children who were followed up, 12 (3.3%) were hospitalised, as reported by caregivers. One of these patients was referred by the HCP at the time of enrolment, five were referred later, either by the same PHC facility during another visit or through another health facility, and six patients were brought in for admission by their families on their own. Two deaths were reported in these hospitalised patients. Most admissions (n = 10, 83.3%) occurred among the younger age group. The majority of patients (n = 10, 83.3%) were admitted for two to four days and were treated in a public healthcare facility. During admission, ceftriaxone, a third-generation cephalosporin, was the most frequently used antibiotic, either alone (25.0%) or in combination with other agents, mostly with amoxicillin (25.0%). In two instances (16.8%), the specific antibiotics administered were not reported by the caregivers. Exploratory analyses of nutritional status and outcomes showed that children with severe undernutrition (WAZ<–3 or WLZ<–3) appeared more frequently among those who experienced hospitalisation. Due to the small number of adverse outcomes, robust statistical modelling was not possible. However, this pattern aligns with established evidence that undernutrition increases pneumonia severity and risk of poor outcomes (Table S2 and S3 in the **Online Supplementary Document**).

At the time of enrolment, out of 373 cases, 350 (93.8%) children were prescribed oral antibiotics. The majority of these cases were prescribed oral amoxicillin 322 (92.0%). Of the 350 cases, six were lost to follow-up (excluding one case that was prescribed home care only). Among the remaining 344 children, caregivers initiated the prescribed oral antibiotics in 307 (89.2%), and 37 (10.8%) did not start taking them (**Table 1**; Table S3 in the **Online Supplementary Document**).

**Figure 3:**
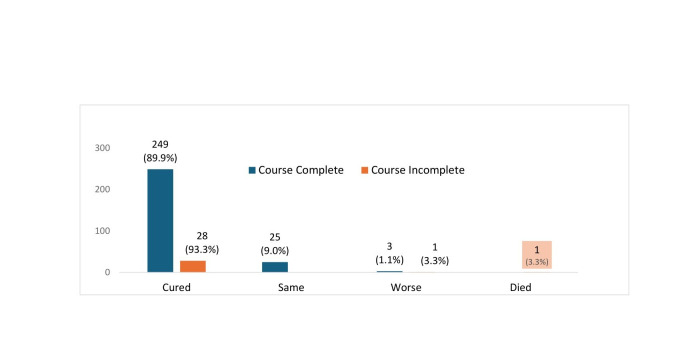
Outcome of children who initiated antibiotic course, course complete versus incomplete (n = 307).

Out of the 307 cases that initiated the treatment, 153 (49.8%) children received antibiotics free of cost at the time of enrolment from the PHC facilities, while 150 (48.9%) bought the medicine from their own pockets, and 4 (1.3%) already had the same prescribed medicine available at home. Overall, 277 of the 307 who initiated antibiotics completed the course, of whom 249 (89.9%) were cured. Of the 30 children whose antibiotic course was not completed, 28 (93.3%) were reportedly cured at the time of follow-up, one child had his health worsened, and one child died (**Figure 3**). Regarding the type of antibiotic prescribed, oral amoxicillin was advised at the time of enrolment to 287 (94.4%) of the 307 who initiated treatment, of whom 261 (91.0%) completed the course, and 235 of these 261 (90.0%) were cured (**Table 3**).

**Table 3:** Cross-tabulation of oral antibiotic types and clinical outcome among children who initiated treatment, stratified by completion of antibiotic course (n = 307), n (%)

Outcome	Oral amoxicillin (n = 287)		Other oral antibiotics (n = 20)*	
	**Course completed (n = 261)**	**Course incomplete (n = 26)**	**Course completed (n = 16)**	**Course incomplete (n = 4)**
Cured	235 (90.0)	24 (92.4)	14 (87.5)	4 (100.0)
Same	23 (8.9)	0 (0.0)	2 (12.5)	0 (0.0)
Worse	3 (1.1)	1 (3.8)	0 (0.0)	0 (0.0)
Died	0 (0.0)	1 (3.8)	0 (0.0)	0 (0.0

*Other medicines that were prescribed: oral co-amoxiclav, oral cefaclor, oral cefixime, oral clarithromycin, oral azithromycin and tab trimethoprim and sulfamethoxazole.

## DISCUSSION

In this prospective observational study from Islamabad and Rawalpindi districts, Pakistan, only two deaths (CFR = 0.6%) were reported among 2–59-month-old children presenting with chest indrawing pneumonia to PHC facilities treated with oral antibiotics on an outpatient basis. Both deceased children were younger than 1 year and had been hospitalised. One died in the hospital, and the other was in transit to the hospital. The majority of children (92.0%) received oral amoxicillin. The caregivers initiated treatment at home for a vast majority of the enrolled children (89.2%). Most of these children (90.0%) were reportedly cured by day 15 of enrolment. Further, 12 children (3.3%) were hospitalised; only one was referred at the time of enrolment, while 11 sought advanced medical care later due to an unchanged or worsening condition.

Our CFR is comparable to that reported in similar observational outpatient-based studies from Ethiopia (0.6%) and Thatta, Pakistan (0.4%), and a community-based study from Homabay, Kenya (0.3%) [20–22]. In contrast, no deaths or hospitalisations were observed in a similar study conducted in Zambia [23]. Likewise, two observational outpatient-based studies, the Multicentre Amoxicillin Severe Pneumonia Study (MASS) from Bangladesh, Egypt, Ghana, and Vietnam, and another from Papua New Guinea, did not report any deaths from chest indrawing pneumonia treatment with oral amoxicillin in outpatient settings [24,25]. This difference in CFR was probably due to regular follow-ups during the first week of therapy and the exclusion of all malnourished children in these studies. Our data are also similar to the <0.5% CFR in children with chest indrawing pneumonia treated with oral amoxicillin reported by three community-based randomised controlled trials (RCTs) [26–28] and four hospital-based RCTs conducted in Africa and Asia [29–32]. Younger infants are more vulnerable to poor outcomes such as treatment failure, hospitalisation, or death, as seen in our study and reported by others [24,33–35].

Our treatment success rate is also comparable to the observational outpatient-based studies from Thatta, Pakistan (96.0%), Ethiopia (97.3%), the MASS study (91.0%), and the Papua New Guinea study (95.0%), indicating that outpatient treatment with oral antibiotics in resource-constrained environments is practical and possible with the provision of appropriate resources [20,21,24,25]. Encouragingly, in our study, over 90.0% of children were prescribed oral antibiotics, primarily amoxicillin, whereas in Thatta, Pakistan, Ethiopia, and Zambia, all patients were prescribed oral antibiotics [20,21,23]. Oral amoxicillin was prescribed to all patients in Ethiopia, 84.0% in Zambia and only 62.0% in Pakistan [20,21,23]. The prescribed treatment initiation in our study was 89.2%, whereas in the Ethiopian study, it was 94.6%, possibly reflecting the impact of structured antibiotic adherence counselling provided to caregivers in that setting [21].

A systematic review of studies from LMICs noted that oral amoxicillin was the most commonly used antibiotic reported in home-based management of chest indrawing pneumonia [33,36]. This widespread preference for oral amoxicillin supports its continued use as a first-line therapy for community-acquired pneumonia in outpatient settings, mainly due to its efficacy, ease of administration, safety, and cost-effectiveness [24,28,37]. The consistency of its use across diverse contexts reinforces the WHO’s recommendation to manage pneumonia without danger signs at the community or outpatient level with oral amoxicillin [38]. It is essential to educate mothers on how to administer home antibiotic treatment, recognise the warning signs, and know when to return to a healthcare provider or health facility to ensure successful treatment.

Several factors related to health systems and caregivers likely affected antibiotic adherence and recovery rates in this study. When oral amoxicillin was reliably available at facilities, caregivers were not required to pay out of pocket to start treatment, which helped improve adherence. Conversely, stock-outs at some PHCs required families to purchase medicines privately, which might have contributed to the 10% who did not initiate therapy [39]. Caregiver factors were equally influential. Limited parental knowledge regarding pneumonia severity and the role of oral antibiotics, household financial constraints, and competing caregiving responsibilities might have affected both the timely initiation and completion of treatment [40]. Distance to the health facility and transportation challenges may also explain why some families sought alternative care or delayed seeking help when the child’s condition worsened [41]. Together, these health-system and household constraints provide important context for understanding why antibiotic adherence was high but not universal, and why a small proportion of children deteriorated or required hospitalisation.

Our study observed that trained HCPs referred 12 children with chest indrawing pneumonia who showed no danger signs to a tertiary care hospital (one case at the caregiver’s request) and advised home care for 11 cases without prescribing antibiotics; neither management strategy was in accordance with the IMCI protocol. These actions reflect both the overuse and underuse of antibiotics. Overprescription contributes to antimicrobial resistance [42], whereas underuse in bacterial infections can be fatal [43]. Some caregivers have misconceptions that the injectable antibiotics are stronger and more effective than the oral antibiotics. Hence, they prefer going to HCPs who prescribe a variety of injectable and oral medicines to satisfy the caregivers who believe that quality of care is equivalent to expensive drugs [43,44]. These findings highlight the need to strengthen adherence to the IMCI protocol [43]. Outcomes were substantially better among those who initiated antibiotics than those who did not. Half of the hospital admissions occurred among patients who had not commenced treatment. We believe that a timely initiation of therapy in these cases might have prevented the need for hospitalisation. Furthermore, children who completed the antibiotic course were less likely to experience treatment failure compared to those who did not. In contrast, non-adherence was associated with poorer outcomes, reinforcing the correlation between adherence and recovery and is consistent with data from a community-based RCT [28].

Inappropriate care-seeking practices are also a challenge in many LMIC settings. In our study, only one out of 12 families accepted the referral advice to a hospital, while the remaining 11 sought either no care or inappropriate care. Family and cultural influences, cost, and accessibility issues are common factors contributing to non-adherence to referral advice [45]. Improper or delayed care-seeking resulted in adverse outcomes like death, as reported from several LMICs [46–48]. The WHO developed guidance for sick young infants when referral is not feasible, which shows that with appropriate training and support, frontline health workers can effectively manage possible serious bacterial infection in infants aged 0–59 days at the primary care level using simplified antibiotic regimens, provided there is timely care-seeking by caregivers, which remains critical to ensuring early diagnosis and improved survival outcomes [48,49].

The findings of this study have direct implications for policy and practice, particularly in supporting the outpatient management of chest indrawing pneumonia at the primary care level, a strategy also recommended by WHO [38]. Prioritising outpatient care for clinically stable cases can reduce the burden on hospitals, ease financial and logistical challenges for families, and free up hospital beds for patients with critical illnesses. There is strong evidence that outpatient treatment significantly reduces costs for both healthcare systems and households, especially in resource-limited settings such as Pakistan [50,51]. In this context, outpatient care emerges as a practical and cost-effective strategy for managing chest indrawing pneumonia. Reducing unnecessary hospital admissions also plays a crucial role in preventing hospital-acquired infections. Hospital-acquired infections or healthcare-associated infections remain a significant public health concern, with 136 million cases reported each year globally; 10 million of which occur in Pakistan alone [52]. Managing eligible pneumonia cases outside hospitals not only alleviates financial strain but also reduces the risk of healthcare-associated infections and helps curb the growing threat of antimicrobial resistance (AMR). An estimated five million AMR-associated deaths occur annually, with 4.3 million deaths in LMICs alone [53]. Around 10 million global AMR-related deaths per year are projected by 2050, underscoring the need for robust infection prevention and control strategies [54]. Oral amoxicillin, a simple and effective first-line treatment for chest indrawing pneumonia, should be reliably made available through strengthened supply chains, regular refresher training for healthcare workers, and supportive supervision. Lastly, stewardship interventions aimed at reducing unnecessary antibiotic use – especially broad-spectrum agents – are most impactful in outpatient settings, where non-severe conditions account for the majority of antibiotic exposure [42,53,54]. Strengthening outpatient management not only supports stewardship goals but also contributes to the broader prevention of avoidable infections and resistance.

A key strength of this study was its prospective design and implementation in real-world PHC settings, using a standardised protocol across facilities, thereby enhancing the generalisability of the findings to similar contexts in LMICs. Second, including quantitative outcomes and qualitative insights from verbal autopsies enriched the understanding of barriers to effective care. Finally, the rigorous follow-up strategy (use of call reminders before follow-up visits, detailed household mapping, and active collaboration with community-level health workers to locate families) and high retention rate provide confidence in the validity of outcome data.

However, we acknowledge a few limitations. First, outcomes were based on caregiver-reported symptoms and recovery status, which may be subject to recall or social desirability bias. Second, the absence of diagnostic tools, such as chest radiographs or laboratory confirmation, limits the ability to precisely classify pneumonia severity or aetiology. Still, these are not routinely used and feasible at the PHC level in most LMICs. Third, although verbal autopsies were used to understand the causes of death, the absence of medical records or death certificates restricted our ability to attribute a cause of death definitively. Fourth, because PHCs were selected based on high ARI caseloads, the study sites might not fully represent lower-volume or more remote PHC facilities in the districts. This intentional selection, although necessary to achieve an adequate sample size, might introduce selection bias and limit the generalisability of the findings to settings with different facility capacities or caseload profiles. Although quality assurance procedures were in place, formal inter-rater reliability was not measured, which may have affected the consistency of assessments across PHCs. Additionally, the frequent turnover of healthcare providers at the facilities necessitated repeated engagement and training from the ground up, resulting in human resource strain and increased costs. Finally, our follow-up lasted 15 days in accordance with the multicountry CIPAM protocol; however, this duration may have missed delayed relapses or complications, potentially introducing temporal bias [17].

## CONCLUSIONS

Our findings reinforce the effectiveness of the IMCI protocol in managing chest indrawing pneumonia among children aged 2–59 months using oral amoxicillin in outpatient settings. This approach holds promise for reducing pneumonia-related mortality, enhancing access and coverage of treatment, minimising unnecessary referrals, easing the burden on overstretched referral facilities, and lowering healthcare costs for both the system and families in resource-constrained environments.

## Additional material


Online Supplementary Document
