# Supplementary material for: Assessment of childhood pneumonia management and outcomes at primary healthcare centres in Islamabad and Rawalpindi: an observational cohort study from Pakistan
Source: J Glob Health. 2026 May 22;16:04137. doi: 10.7189/jogh.16.04137 (PMC13196488; doi:10.7189/jogh.16.04137)
Supplement: Online Supplementary Document [file jogh-16-04137-s001.pdf]

Supplement to: Mahmood H, Hafeez A, Kashif A, Sheraz SY, Shehzadi M, Qazi SA, Nisar YB. Assessment of childhood pneumonia management and outcomes at primary healthcare centres in Islamabad and Rawalpindi: an observational cohort study from Pakistan. J Glob Health. 2026;16:04137.

Supplementary Table 1. Stratified outcomes by nutritional status

Nutritional Status (Under-weight, Weight for Age Z-score - WAZ)

Nutritional category	N (total)	Recovered n (%)	Same n (%)	Worsened n (%)	Deaths n (%)	CFR (%)
**Normal (> -2 SD)**	312	275(89.87%)	27(8.82%)	3(0.98%)	1(0.33%)	0.33%
**Moderate (-2 to -3 SD)**	44	37(86.06%)	4(9.30%)	1(2.32%)	1(2.32%)	2.32%
**Severe (< -3 SD)**	17	15(88.24%)	2(11.76%)	0(0.00%)	0(0.00%)	0.00%

Nutritional Status (Stunting, Height for Age Z-score - HAZ)

Nutritional category	N (total)	Recovered n (%)	Same n (%)	Worsened n (%)	Deaths n (%)	CFR (%)
**Normal (> –2 SD)**	278	247 (90.82%)	21 (7.72%)	2 (0.73%)	2 (0.73%)	0.73%
**Moderate (–2 to –3 SD)**	43	39 (90.70%)	4 (9.30%)	0 (0.00%)	0 (0.00%)	0.00%
**Severe (< –3 SD)**	52	41 (80.39%)	8 (15.69%)	2 (3.92 %)	0 (0.00%)	0.00%

Nutritional Status (Wasting, Weight for Height Z-score - WHZ)

Nutritional category	N (total)	Recovered n (%)	Same n (%)	Worsened n (%)	Deaths n (%)	CFR (%)
**Normal (> –2 SD)**	272	235 (88.34%)	27 (10.15%)	3 (1.13%)	1 (0.38%)	0.38%
**Moderate (–2 to –3 SD)**	64	59 (92.19%)	4 (6.25%)	0 (0.00%)	1 (1.56%)	5.56%
**Severe (< –3 SD)**	37	33 (91.67%)	2 (5.55%)	1 (2.78%)	0 (0.00%)	0.00%

Nutritional Status (Mid upper-arm circumference - MUAC)

Nutritional category	N (total)	Recovered n (%)	Same n (%)	Worsened n (%)	Deaths n (%)	CFR (%)
**Normal (≥ 12.5 cm)**	364	320 (89.64%)	31 (8.68%)	4 (1.12%)	2 (0.56)	0.56
**Moderate (11.5–<12.5 cm)**	7	5 (71.43%)	2 (28.57%)	0 (0.00%)	0 (0.00%)	0.00
**Severe (<11.5 cm)**	2	2 (100.00%)	0 (0.00%)	0 (0.00%)	0 (0.00%)	0.00

Percentages are calculated based on valid outcomes, excluding 7 lost to follow-up.

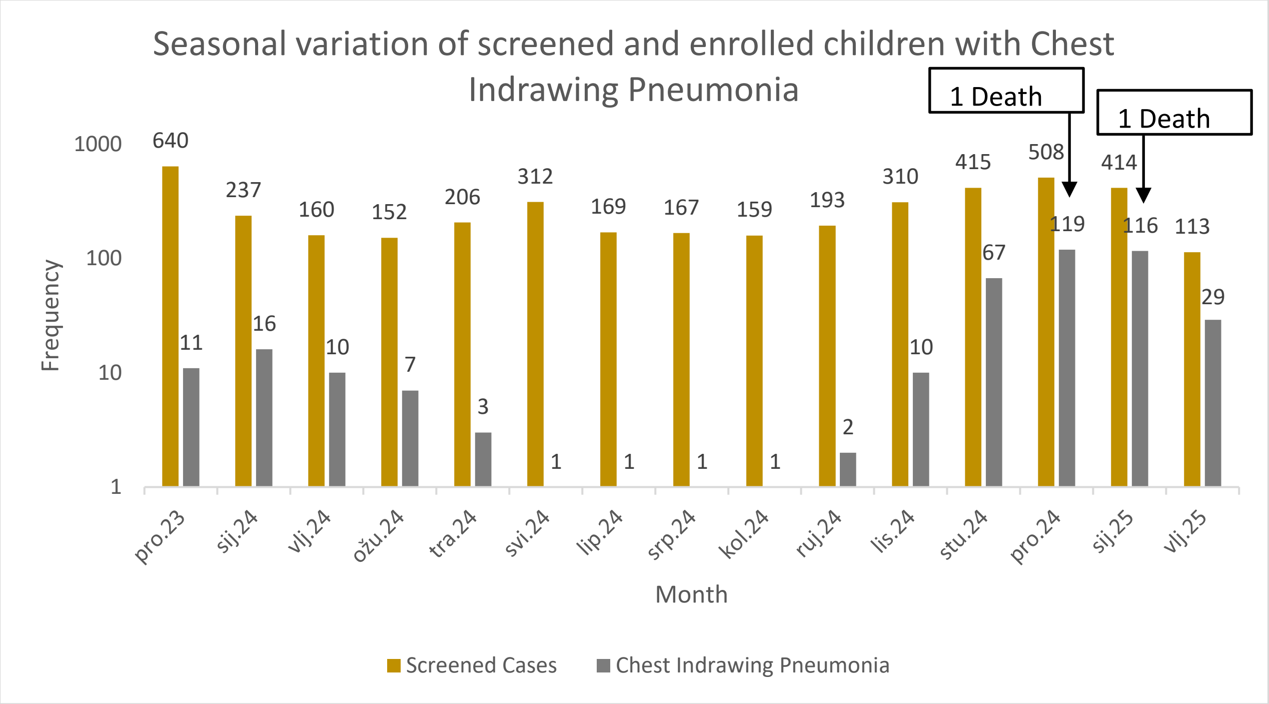

Supplementary Figure 1: Seasonal variation of screened and enrolled children with chest indrawing pneumonia.

The y-axis is presented on a logarithmic scale to enhance the visual clarity of the data, given the wide variation in frequencies between screened cases and confirmed chest indrawing pneumonia cases across the study period.
